# Getting More Out of Biomedical Documents with GATE's Full Lifecycle Open Source Text Analytics

**DOI:** 10.1371/journal.pcbi.1002854

**Published:** 2013-02-07

**Authors:** Hamish Cunningham, Valentin Tablan, Angus Roberts, Kalina Bontcheva

**Affiliations:** 1 Department of Computer Science, University of Sheffield, Sheffield, United Kingdom; UCSD, United States of America

## Abstract

This software article describes the GATE family of open source text analysis tools and processes. GATE is one of the most widely used systems of its type with yearly download rates of tens of thousands and many active users in both academic and industrial contexts. In this paper we report three examples of GATE-based systems operating in the life sciences and in medicine. First, in genome-wide association studies which have contributed to discovery of a head and neck cancer mutation association. Second, medical records analysis which has significantly increased the statistical power of treatment/outcome models in the UK's largest psychiatric patient cohort. Third, richer constructs in drug-related searching. We also explore the ways in which the GATE family supports the various stages of the lifecycle present in our examples. We conclude that the deployment of text mining for document abstraction or rich search and navigation is best thought of as a process, and that with the right computational tools and data collection strategies this process can be made defined and repeatable. The GATE research programme is now 20 years old and has grown from its roots as a specialist development tool for text processing to become a rather comprehensive ecosystem, bringing together software developers, language engineers and research staff from diverse fields. GATE now has a strong claim to cover a uniquely wide range of the lifecycle of text analysis systems. It forms a focal point for the integration and reuse of advances that have been made by many people (the majority outside of the authors' own group) who work in text processing for biomedicine and other areas. GATE is available online <1> under GNU open source licences and runs on all major operating systems. Support is available from an active user and developer community and also on a commercial basis.

This is a *PLOS Computational Biology* Software Article

## Introduction

We talk, we write, we listen or read, and we are so skilled in our use of language that we are seldom aware of the complexities involved in its production and consumption. It is natural, therefore, that a large proportion of what we know of the world is externalised exclusively in textual form. That fraction of our science, technology and art that is codified in databases, taxonomies, ontologies and the like (let's call this *structured data*) is relatively small. Structured data is, of course, machine-tractable in ways that text can never be (at least in advance of a true artificial intelligence, something that recedes as fast as ever over the long-term horizon). Unfortunately structure can also be inflexible and expensive to produce in ways that text is not.

When scientific results are delivered exclusively via textual publication, the process of replicating these results is often inefficient as a consequence. Although advances in computational platforms raise exciting possibilities for increased sharing and reuse of experimental setups and research results, still there is little sign that scientific publication will cease its relentless growth in the near future.

Similarly, although clinical recording continues to make progress away from paper and towards on-line systems with structured data models, still the primacy of text as a persistent communication mechanism (within and between medical teams and between medics and their patients) means that medical records will contain a wealth of textual, unstructured material for the forseeable future.

Technology seeks to bridge this gap under the headings of text mining, or natural language processing (NLP), with biomedical text mining and BioNLP being the subfields related to biomedicine. Cohen and Hunter [1], Rzhetsky *et al.* [2] and Rodriguez-Esteban [3] provide introductions to the topic; the general aim is to discern the semantic content of text and encode this in a structured way, often by adding annotations to segments of the text. An example: having created an ontology (or database) of gene names, with each gene having a unique identifier, then a relevant document would be annotated such that all occurrences of (often ambiguous) gene names in the text are annotated with the correct unique identifier.

This paper introduces a research programme (now 20 years old) that has resulted in GATE, a General Architecture for Text Engineering [4], [5]. In recent years GATE has grown from its roots as a specialist development tool for text processing to become a rather comprehensive ecosystem bringing together software developers, language engineers and research staff from diverse fields. GATE now has a strong claim to cover a uniquely wide range of the lifecycle of text analysis systems. It forms a focal point for the integration and reuse of advances that have been made by many people (the majority outside of the authors' own group) who work in text processing for biomedicine and other areas.

In line with the trends towards openness in life sciences R&D and in publishing, GATE is 100% open source. This brings benefits that have been recognised elsewhere (vendor independence; security; longevity; flexibility; minimisation of costs; see e.g. [6], [7]). Less often remarked upon but arguably particularly significant in medical contexts are traceability and transparency. Findings that are explicable and fully open may be worth more than results that appear magically (but mysteriously) from black boxes.

In this paper we will discuss several areas within biomedicine where GATE has facilitated advances. First, in providing evidence in genome-wide association studies, resulting in the finding of a new gene/disease association for head and neck cancer. Second, finding data in medical records, allowing a significant amount of information to be added to the evidence base for clinical planning and policy formation. Third, in creating new search functionality in drug-related literature search.

We begin by describing the technology that has been used in these applications, before describing each of the projects in more detail.

## Design and Implementation

### Summary

The GATE family of tools has grown over the years to include a desktop application for developers, a collaborative workflow-based web application, an index server, a Java library, an architecture and a process. To summarise, GATE comprises:

- **GATE Developer**: an integrated development environment (IDE) for language processing components, which is bundled with a widely used information extraction [8] system and a diverse set of several hundred other plugins <2>;
- a cloud computing solution for hosted large-scale text processing, **GATE Cloud** <3>;
- **GATE Teamware**: a collaborative environment for large-scale manual semantic annotation projects built around a workflow engine and a heavily-optimised backend service infrastructure;
- a multi-paradigm index server, **GATE Mímir**, which can be used to index and search over text, annotations, semantic schemas (ontologies), and semantic meta-data (instances), allowing queries that arbitrarily mix full-text, structural, linguistic and semantic constraints and that can scale to terabytes of text;
- a framework, **GATE Embedded**: an object library optimised for inclusion in diverse applications giving access to all the services used by GATE Developer and others;
- an architecture: a high-level organisational picture of language processing software composition;
- a process for the creation of robust and maintainable services <39>;
- a wiki <40> (mainly as host for our own web content, but also as a vehicle for an experimental programme in controlled natural languages [9]).

(Note that GATE Developer and Embedded are bundled, and in early distributions were referred to just as ‘GATE’.)

### Background

The GATE family is intended to minimise time and effort in developing and maintaining rich information extraction, retrieval and management systems, while staying at or near to the state of the technological art, partly by favouring interoperation and reuse over reinvention.

Our programme originated in the early 1990s, partly as a response to research in software reuse and in object-oriented design methods and programming languages [10]. The first phase of our work was to analyse a wide range of the approaches taken to software architecture in the field of natural language processing [11]–[13]. We used this analysis to propose a high level abstraction of how language processing software systems can be composed so as to maximise reusability, both of the engineering functions underlying these systems and of new instances of particular cases. This model (or architecture) made particular use of work on interoperation of information extraction systems [14] and work on stand-off markup in XML processing pipelines [15]. The graph-based appoach that we (and others) adopted has since become a defacto standard [16] and underlies the OASIS/Open UIMA standard [17]. (Standoff markup in XML [18], [19] is an important and common case, as are more explicitly graph-oriented systems such as GATE, ATLAS or UIMA [4], [20], [21] – see below.)

In parallel with this analysis and design process, we developed two related systems, GATE Developer and GATE Embedded, which this section will detail, along with later arrivals GATE Cloud and Mímir. (For details of GATE Teamware see <42> or [22].)

The closest comparable system to GATE is UIMA <38> [20], which provides a library which is similar to the core of GATE Embedded (but with a more explicit type system). UIMA also provides some graphical facilities for running analysis pipelines that are a subset of some of those in GATE Developer. Finally, there is a scaling tool, UIMA Asynchronous Scaleout, which provides a subset of the some of the services of GATE Cloud. GATE and UIMA are complementary, and we have developed an interoperation layer that will run UIMA-based applications within GATE and vice-versa. An interoperation mechanism based on the GrAF format [23] is also available.

### GATE Developer

GATE Developer is a specialist Integrated Development Environment (IDE) for language engineering R&D. It is analogous to systems like Eclipse or Netbeans for programmers, or Mathematica or SPSS for mathematics or statistics work. The system performs tasks such as:

- Visualisation and editing of domain-specific data structures associated with text: annotation graphs, ontologies, terminologies, syntax trees, etc.
- Constructing applications from sets of components (or plugins).
- Measurement, evaluation and benchmarking of automatic systems relative to *gold standard* data produced by human beings, or to previous runs of variants of experimental setups.

A sophisticated graphical user interface provides access to the models of the GATE architecture and particular instantiations of that architecture.

**Figure 1:**
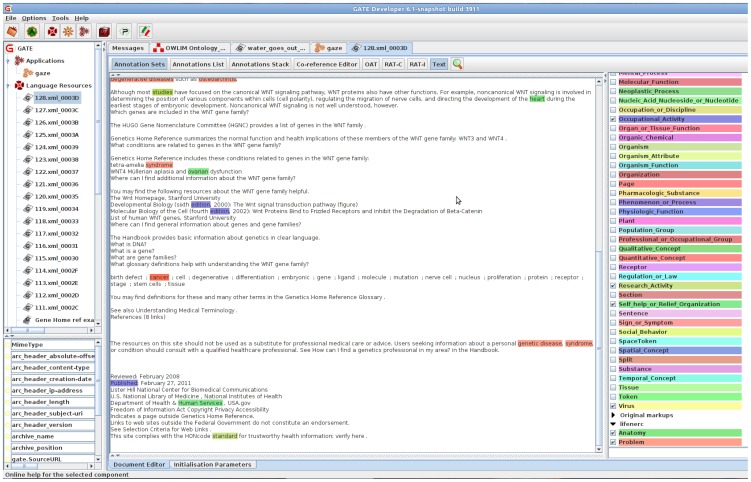
The GATE developer interface.

Figure 1 displays analysis results over a page from the Genetics Home Reference website <4>. The central pane shows a version of the source text from which formatting markup has been removed (and converted into arcs in an annotation graph associated with the document). The left panes detail resources loaded in the system, including the application being used to annotate the text (with biomedical named entities in this case) and the documents under analysis. The right pane lists the types of annotation that have been applied to the document (for example anatomical locus or tissue type). The central pane responds to selection of annotation types with various forms of highlighting and other visualisations.

### GATE Embedded

Underlying GATE Developer (and most of our other systems) is an object-oriented Java framework called GATE Embedded. Some of the architectural principles which we adopted when developing the framework are as follows:

- Neutrality. The framework tries hard to be non-prescriptive and theory neutral. This is a strength because it means that no approach to language processing that users favour is excluded, but it is also a weakness because more restricted and specialised tools can capture more abstractions about their target domains, hence:
- Re-use. We minimise the impact of that weakness by emphasising re-use and interoperation with related systems, and avoiding reimplementation wherever possible. Thus we provide diverse XML support, integration with the Protégé ontology editor [24], the OWLIM semantic repository [25], the Weka machine learning library [26], the Lingpipe <36> and OpenNLP <37> language analysis pipelines, ABNER [27], MetaMap [28], GENIA [29], AbGene [30], BioTagger [31], LinkedLifeData <13>, and the SVM Lite library [32], to name but a few. (More details on the specifically biomedical members of this set appear below.)
- Componentisation. Almost everything in GATE is modelled as a component, and the various component sets are all user-extendable. This means that all of the functions of the system can be swapped out, extended or replaced by users and developers with specific needs.
- Multiple usage modes. Almost all operations are available both from API (GATE Embedded) and UI (GATE Developer). A common process is to develop and test using the IDE and then embed in the target environment using the Java library. In both cases exactly the same underlying framework is in operation.

The set of plugins that are integrated with GATE is called CREOLE, a Collection of REusable Objects for Language Engineering. Components are defined as Java Beans bundled with XML configuration, and the overheads imposed by the model are very small (the minimal component comprises a few lines of Java code plus a few lines of XML). Components can be packaged in the same way as other Java libraries and can be loaded over the network via a URL.

**Figure 2:**
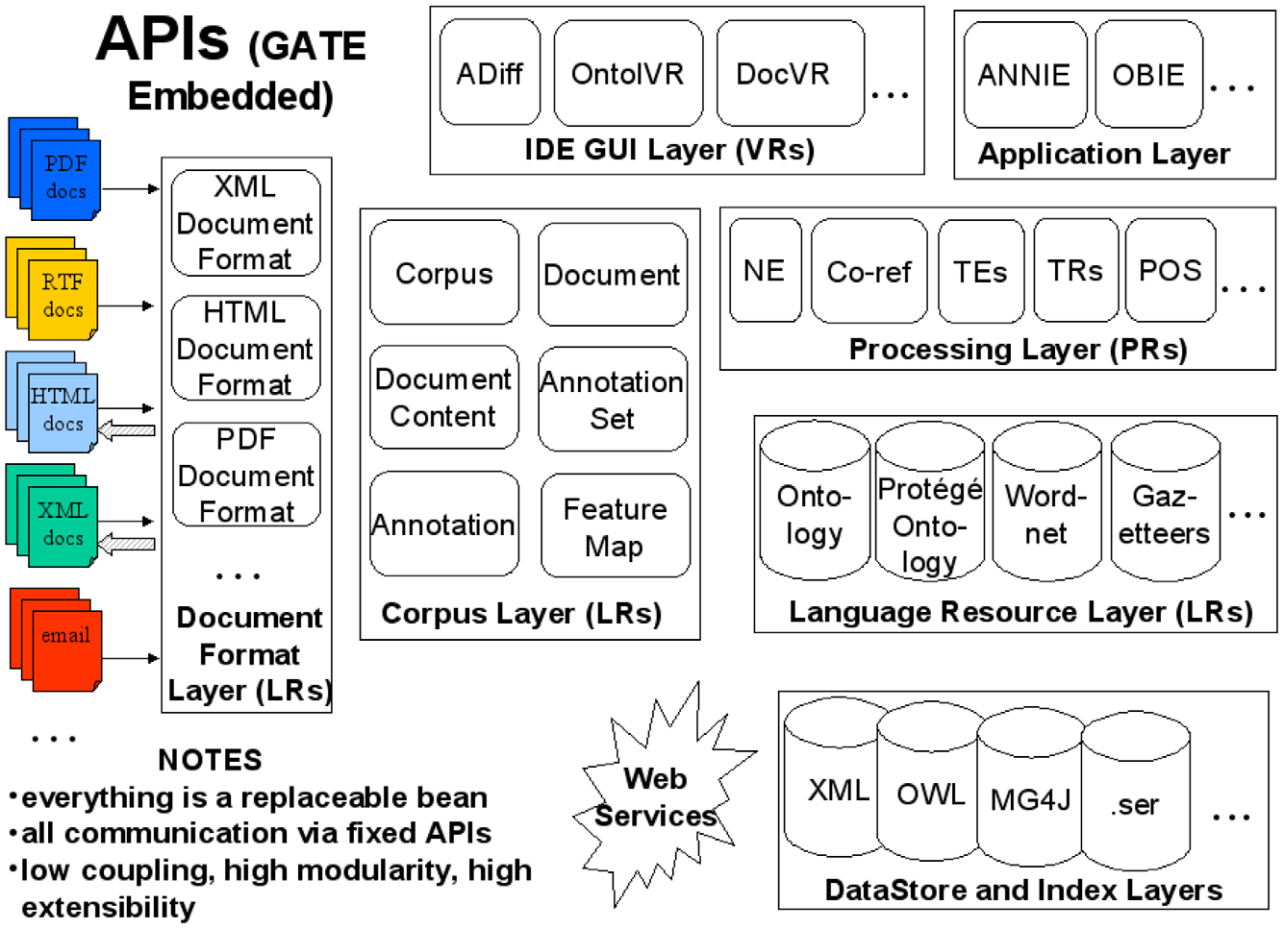
GATE embedded APIs. GATE provides a set of Java APIs, called GATE Embedded. This figure summarises the modules provided. Language resources (LRs) are data-only resources such as lexica, corpora or ontologies. Processing Resources (PRs) are principally programmatic or algorithmic. Visual resources (VRs) allow users to interact visually with other resources.

GATE Embedded encapsulates a number of modular APIs for text processing, which are summarised in Figure 2.

These APIs cover functions including:

- persistence, visualisation and editing
- a finite state transduction language (JAPE, a Java Annotation Patterns Engine [33])
- extraction of training instances for machine learning (ML – methods for automated abstraction of pattern recognition models from data, see e.g. [34])
- pluggable ML implementations (e.g. Weka, [26], support vector machines [32], etc.)
- components for language analysis, e.g. parsers, taggers and stemmers for various languages
- a very widely used information extraction system (ANNIE) which has been evaluated in comparative events including MUC, TREC, ACE, DUC, Pascal, NTCIR, etc. [35]–[39]
- indexing and search tools (including Lucene, Google and Yahoo plugins)
- a simple API for RDF, OWL and Linked Data

The modularity of the library and the low level of commitment imposed on its clients has proven flexible enough to prosper for more than a decade since the release of version 2 (the first Java version).

### GATE Cloud

As long as a decade ago a research team at Merck KGaA pharmaceuticals ran GATE in a 100-node cluster to process MEDLINE abstracts. More recently companies like Amazon began selling computing capacity in the form of Cloud Computing (detailed in this journal by Fusaro *et al.* [40]).

We have developed a service at GATECloud.net [41] <3> which deploys GATE analysis pipelines and GATE server products on Amazon EC2 (Elastic Compute Cloud – a popular cloud computing platform). GATE annotation pipelines provide a PaaS (Platform as a Service [42]) arrangement: software produced using GATE Developer/Embedded can be trivially scaled up to large data volumes. In this way GATE Teamware and Mímir on the cloud provide a SaaS (Software as a Service) arrangement where responsibility for installation and administration are removed from the end user.

GATE Cloud is based on a **parallel** execution engine of automatic annotation processes (using pooling and model sharing to minimise the load on individual nodes) and **distributed** execution of the parallel engine [41]. Its characteristics include:

- **scalability**: auto-scaling of processor swarms dependent on loading;
- **flexibility**: user-visible parameters configure system behaviour, select the GATE application being executed, the input protocol used for reading documents, the output protocol used for exporting the resulting annotations, and so on;
- **robustness**: jobs run unattended over large data sets using a parallelisation system that has been extensively tested and profiled.

Any errors and exceptions that occur during processing are trapped and reported, and if the process crashes (e.g. due to hardware failure), upon restart it will resume execution where it left off. Some functionality is similar to that of more general purpose systems such as Hadoop [43], but this is not currently used.

### GATE Mímir: A Multi-Paradigm Index Server

Consider the following three types of information retrieval systems:

- full-text-based, with boolean and proximity operators [44];
- annotation-based, with an underlying graph representation encoding structured information about text ranges [45];
- ontology-based, with hierarchical conceptual schemas plus concept instance sets from documents and databases [46].

Systems for high-value content retrieval are likely to combine elements of all three styles, posing difficult problems of representation, persistence, indexing and querying. Mímir (meaning ‘the rememberer, the wise one’ in Old Norse) is a Multi-paradigm Information Management Index and Repository [47] which can be used to index and search over text, annotations, semantic schemas (ontologies), and semantic meta-data (instance data). It allows queries that arbitrarily mix full text, boolean, structural, linguistic and semantic queries and can scale to terabytes of text.

The systems that Mímir supports pose three quite different sets of requirements for persistence and efficient indexing, search and access:

- **Augmented full text.** Having extracted information from documents, we then need to support the types of boolean full text queries that are familiar from large numbers of conventional search systems [44], [48], [49].
- **Annotation graphs.** These structures consist of nodes which are offsets into textual documents, linked by arcs holding type names and bundles of attribute/value pairs. It is important to note that the data is graph-structured, so when serialising to XML mechanisms that are external to the markup tree have to be employed (often referred to as ‘stand-off markup’). Therefore XML persistence and query mechanisms (such as those based on XQuery or XPath) have not addressed the graph indexing problem.
- **Ontology and Knowledge Base.** Finally, when we extract information in relational or hierarchical forms we structure the schema using an ontology language (and tend to call the result a ‘knowledge base’, or KB). The ontology represents the data schema and comprises a hierarchy of class types and a hierarchy of properties that are applicable to instances of classes. The instance data represents facts that are known to the systems and is typically at least partially derived from semantic annotation over documents. KB data is used to reach a higher level of abstraction over the information in the documents which enables conceptual queries such as ‘find all mentions of drugs that contain acetylsalicylic acid’.

The first and last of these problems were relatively easy to solve. We use MG4J <5> [50] for full text indexing, and we use OWL stored in the OWLIM semantic repository [25] to represent and query ontological data (via SPARQL, a standard query language for ontological data [51]). Indexing and querying annotation graphs is an indexing task which has not been widely treated, and to this we now turn.

**Figure 3:**
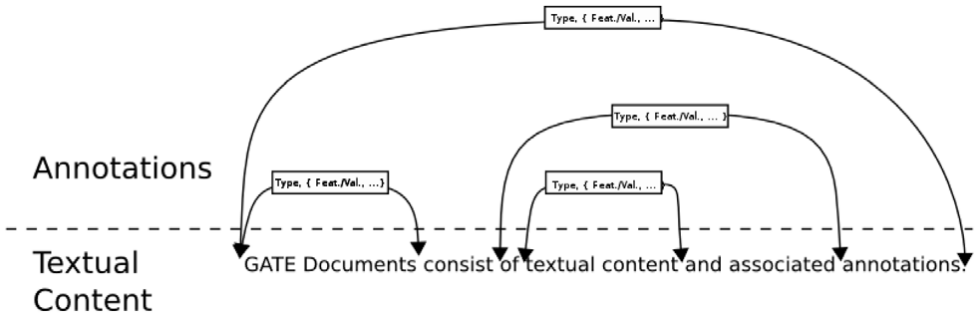
An annotation graph. In GATE, annotations are encoded by associating features with character offsets, indicating the text to which they pertain.

Annotation graphs associate arbitrary feature/value pairs (arcs) with character offsets in text (nodes). An example is shown in Figure 3.

**Figure 4:**
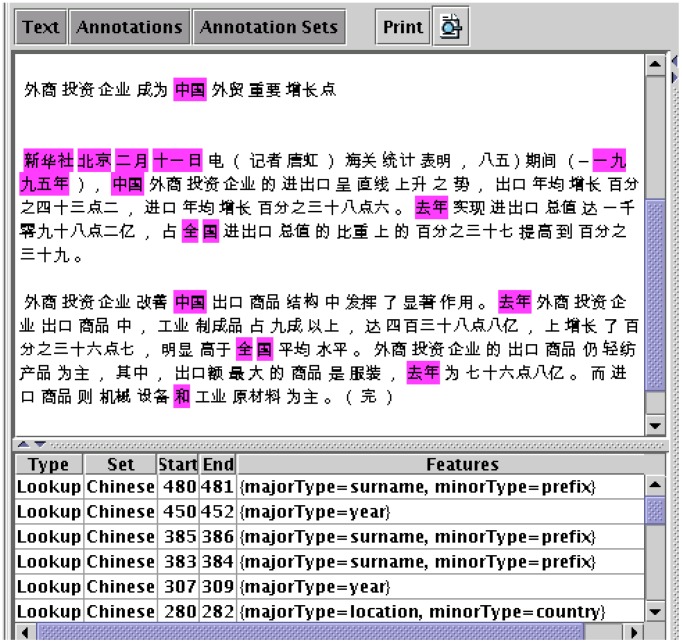
Chinese annotations. In GATE's document view, annotations are shown as highlighted sections of text. This figure shows Chinese text with highlighted annotations. The annotations are listed at the bottom, showing their type, offsets and features.

GATE Embedded uses these graphs as its native format for language analysis data, and GATE Developer provides visualisation and editing facilities for the graphs. For example, Figure 4 shows a document view showing highlighting of particular annotation types and a list view of the details of those annotations (start and end offsets, type, and bundle of feature/value pairs).

Two additional system features are relevant to the problem of indexing and searching annotation data:

- First, GATE includes a finite state transduction language called JAPE (Java Annotation Patterns Engine) that defines a rich regular expression language ([52] – a popular and efficient pattern recognition technique) for matching within annotation graphs.
- Second, GATE Developer includes ANNIC (ANNotations In Context), a visualisation tool inspired by the KWIC (Key Words In Context) tools that have long been a staple of the lexicographer's toolbox.

**Figure 5:**
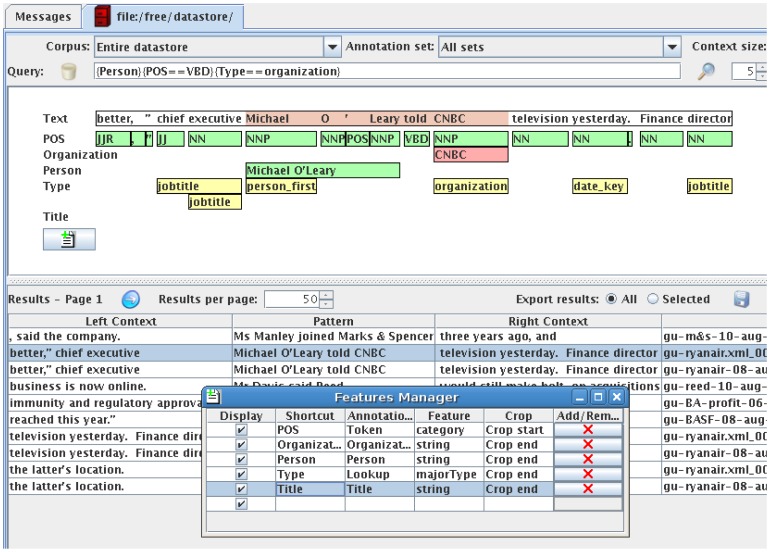
ANNIC (ANNotations In Context). Complex queries are supported, such as a query that searches for person annotations followed by past tense verbs followed by organisation names, as shown in this figure. The query appears in the third line from the top; the patterns described are for people annotation followed by organisation annotations. All matching text ranges then appear in the lower half of the tool, with a graphical representation of the individual annotations concerned in the middle part.

The two features come together to a degree in that ANNIC allows queries using a JAPE-like language. For example, a query that searches for person annotations followed by past tense verbs followed by organisation names is shown in Figure 5.

The challenge that we faced when trying to generalise ANNIC to indexes in the gigabyte to terabyte range was scaling. Our initial implementation (based on Lucene [53]) generated an index disk footprint on the order of exponential in relation to the source data, and therefore could not scale beyond very small data sets. In analysing the problem we considered a range of existing solutions from the XML, RDBMS and augmented full text indexing fields and solicited input from each of these communities at a workshop in May 2008 on *Persisting, Indexing and Querying Multi-Paradigm Text Models*, at the Information Retrieval Facility <43> in Vienna. Our discussions failed to identify a pre-existing solution that could be applied directly (XML indexing and retrieval is biased towards trees; relational databases are biased towards relations) but we did discover that the implementation of sequence operators (a mechanism for representing longer structures than is typical in word-level indexing systems) in MG4J [50] was sufficiently efficient to represent a possible solution, and this is how we implemented the annotation graph support in Mímir.

**Figure 6:**
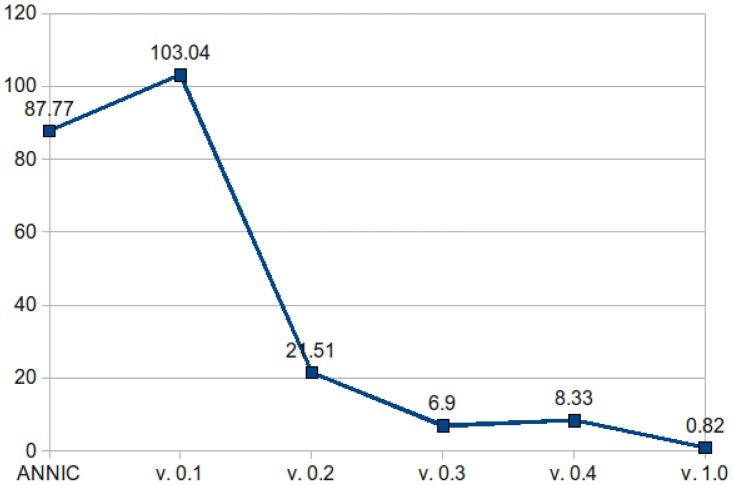
Mímir index size. As this figure shows, in later versions of Mímir, software improvements meant that the index could be reduced in size, allowing much larger document collections to be indexed.

This implementation scaled well. For example we reduced the disk footprint of the indices as shown in Figure 6. In the figure, the X axis is the various versions over time, starting with our ANNIC baseline; the Y axis is disk footprint size. This allowed us to index document collections in the tens of gigabytes. To scale up to the terabyte range we implemented index federation, whereby document sets are partitioned, queries fired against multiple indices and the results combined. Incremental indexing (the ability to add to an index after its initial creation) is in development.

### Biomedical GATE Components and the Lifecycle

We conclude the first half of the paper with a look at GATE components that are specific to biomedicine and at how the various members of the GATE family contribute support to text analysis lifecycles.

### Biomedical Components

Documents from the biomedical domain offer a number of challenges, including a highly specialised vocabulary, words that include mixed case and numbers requiring unusual tokenization as well as common English words used with a domain specific sense. Many of these problems can only be solved through the use of domain specific resources.

Many GATE components can be adapted with little or no effort to help with processing biomedical documents. The Large Knowledge Base Gazetteer (<12> in [5]) can be initialized against a biomedical ontology such as Linked Life Data <13> [54] in order to annotate many different domain specific concepts. The Language Identification resource can also be trained to differentiate between document domains instead of languages, which could help target specific resources to specific documents.

Also many plugins can be used “as is” to extract information from biomedical documents. For example, the Measurements Tagger of [5] can be used to extract information about the dose of a medication, or the weight of patients in a study.

The rest of this section, however, documents the resources included with GATE which are focused purely on processing biomedical documents.

**ABNER** is A Biomedical Named Entity Recogniser [27]. It uses machine learning (linear-chain conditional random fields – CRFs) to find entities such as genes, cell types, and DNA in text. The tagger finds and annotates entities of the following types: Protein; DNA; RNA; CellLine; CellType. ABNER does support training of models on other data, but this functionality is not, however, supported by the GATE wrapper. For further details please refer to the ABNER documentation at <26>.

**MetaMap** (from the National Library of Medicine) maps biomedical text to the UMLS Metathesaurus and allows Metathesaurus concepts to be discovered in a text corpus [28] <33>.

#### Gspell biomedical spelling suggestion and correction

This plugin wraps the GSpell <27> API, from the National Library of Medicine Lexical Systems Group, to add spelling suggestion annotations. The GSpell plugin has a number of options to customise the behaviour and to reduce the number of false positives in the spelling suggestions. For example, ignore words and spelling suggestions shorter than a given threshold, and regular expressions to filter the input to the spell checker. Two filters are provided by default: ignore capitalised abbreviations/words in all caps, and words starting or ending with a digit.

**BADREX** (identifying *B*iomedical *A*bbreviations using *D*ynamic *R*egular *E*xpressions) [55] is a GATE plugin that annotates, expands and corefers term-abbreviation pairs using parameterisable regular expressions that generalise and extend the Schwartz-Hearst algorithm [56]. In addition it uses a subset of the inner–outer selection rules described in the [57] ALICE algorithm. Rather than simply extracting terms and their abbreviations, it annotates them in situ and adds the corresponding long-form and short-form text as features on each. In coreference mode BADREX expands all abbreviations in the text that match the short form of the most recently matched long-form–short-form pair. In addition, there is the option of annotating and classifying common medical abbreviations extracted from Wikipedia.

#### MiniChem/Drug Tagger

The MiniChem Tagger is a GATE plugin uses a small set (around 500) of chemistry morphemes classified into 10 types (root, suffix, multiplier etc), and some deterministic rules based on the Wikipedia IUPAC entries, to identify chemical names, drug names and chemical formula in text. The plugin can be downloaded from <28>.

#### AbGene

Support for using AbGene [30] (a modified version of the Brill tagger), to annotate gene names, within GATE. AbGene can be downloaded <34>.

#### GENIA

A number of different biomedical language processing tools have been developed under the auspices of the GENIA Project <29>. Support is provided within GATE for using both the GENIA sentence splitter and the tagger, which provides tokenization, part-of-speech tagging, shallow parsing and named entity recognition. For more details on the GENIA tagger and its performance over biomedical text see [29].

**The Penn BioTagger** software suite <35> provides a biomedical tokenizer and three taggers for gene entities [31], genomic variations entities [58] and malignancy type entities [59]. All four components are available within GATE via the Tagger_PennBio plugin.

**MutationFinder** <30> is a high-performance IE tool designed to extract mentions of point mutations from free text [60]. A point mutation, or single base substitution, is a type of mutation that causes the replacement of a single base nucleotide with another nucleotide of the genetic material, DNA or RNA. In a blind test data, MutationFinder achieved a precision of 98.4% and a recall of 81.9% when extracting point mutation mentions.

**NormaGene** <31> is a web service, provided by the BiTeM group <32> in Geneva. The service provides tools for both gene tagging and normalization, although currently only tagging is supported by this GATE wrapper.

**Linked Life Data (LLD**, <13> [54]) is an aggregation of several existing taxonomic and terminological resources for life sciences represented in the OWL ontology language [61]. (Sources include: Uniprot, Entrez-Gene, iProClass, the Gene Ontology, BioGRID Complete, the NCI Pathway Interaction Database, the Cancer Cell Map, Reactome, BioCarta, KEGG, BioCyc, the NCBI Taxonomy.) Several resources are modelled using schemata from the BioPAX data exchange language [62]. The outcome is a means to access all the resources via a single mechanism. A key challenge for such aggregated data services is performance – the data involved is in the billions of statements – but LLD scales well to these sizes via the underlying semantic repository, which is specifically optimised for the large scale.

**Organism Tagger** [63] report a tagger for species names, ‘a useful step for many other analysis tasks; in particular it provides for species-specific queries to the literature and can help in disambiguating other biological entities in a document, such as proteins’ according to the authors, and uses a GATE analysis pipeline. This pipeline identifies species, their genus and strain parts, and normalises forms such as abbreviations and acronyms to the organisms normal scientific nomenclature. The normalised form is then matched against the NCBI Taxonomy Database, adding a URL to its web page. More details: <41>.

### The Text Analysis Lifecycle

As discussed in the introduction, text analysis projects typically follow certain patterns, or lifecycles. A central problem is to define the extraction task with sufficient precision that human annotators can perform the task with a high level of agreement (this level represents a ceiling to machine performance) and to create high quality example data with which to drive development and measurement of the automatic analysis pipeline. It is common to use double or triple annotation, where several people perform the extraction task independently and we then measure their level of agreement (the *Inter-Annotator Agreement*, or IAA) to quantify and control quality of this data.

To summarise the process, the steps that typically compose the text analysis lifecycle (and the GATE tools that are relevant at each step) are as follows:

1. Aggregate the text collection that you need to provide additional access to, or abstraction over (scientific papers, patient records, technical reports, clinical trials documents, emails, tweets, transcripts, blogs, comments, acts of parliament, and so on and so forth). This is the *corpus* or collection of *corpora* for the project.
2. Develop a structured description of interesting things in the text. This may be as simple as a corporate telephone directory, or a set of drug names, or a chemical taxonomy, or something from the Linked Data cloud [64], or from Linked Life Data <13>. This forms the *ontology* for the project.
3. Specify the extraction task and verify the specification. Use GATE Teamware (or, for small projects, GATE Developer) to manually mark up a *gold standard* example set of annotations of the corpus (1.) relative to the ontology (2.). (Inter-Annotator Agreement tools help drive refinement of the task specification; bootstrapping tools, where we use a combination of manual and automatic methods, help reduce the cost of the manual work.)
4. Prototype the text analysis pipeline. Use GATE Developer to build a *semantic annotation pipeline* to do the annotation job automatically and measure performance against the gold standard. (If you have enough training data from (3.) or elsewhere you can use Developer's machine learning facilities here.)
5. Deploy and verify the analysis system. Take the pipeline from (4.) and apply it to your corpus using GATE Cloud (or embed it in your own systems using GATE Embedded). Use it to bootstrap more manual (now semi-automatic) quality assurance work in Teamware or Developer.
6. Populate an index server. Use GATE Mímir to store the annotations relative to the ontology in a *multiparadigm index server*.
7. Expose the results to end-users. Either:
  - export the data for analysis in statistics packages, databases, etc., or:
  - write a domain-specific user interface to go on top of Mímir, or integrate it in your existing front-end systems via Mímir's RESTful web APIs.

Certain steps or sequences of steps are often iterated in the manner of agile development methods, and integral testing also mirrors agile practice [65], [66].

The end result is search (or abstraction) that applies your annotations and your ontology to your corpus, but the software products are only part of the outcome. We also attain a robust and sustainable process for maintaining the system and for coping with changing information needs and/or changing text. In each case we use manual or semi-automatic annotation and automated measurement and regression testing to ensure stability of existing analyses or to structure development of new analyses.

## Results

In this section, we give three examples of biomedical problems solved using GATE. Firstly, we show how GATE has been used to adjust association priors using published literature, thus facilitating the discovery of gene associations. Secondly, we show GATE being used to extract data from free text fields in clinical records, making a large amount of new data available for analysis and improving the accuracy and coverage of existing data. Finally, we show how GATE has been used to annotate drug names in patents to provide enhanced search capabilities. These examples cover typical use cases of text analysis: the first two make new abstractions over textual data; the third provides new search and navigation facilities.

### Facilitating Gene-Disease Association Studies

As noted above, we begin with an example which is representative of uses of text analysis to perform abstraction over textual data in order to support some other process – in this case gene-disease association studies.

It has been hypothesised that genetic factors play a strong role in susceptibility to disease, and that in future targeted pharmaceuticals will become available that are tailored to our individual genetic particularities. A substantial body of work has addressed the identification of associations between mutations (usually SNPs – single nucleotide polymorphisms) and diseases. It is hoped that these associations will inform new pharmaceutical interventions against the diseases concerned.

In recent years gene-disease association researchers have often moved from a candidate gene approach (where genes are selected and tested based on prior knowledge and hypotheses) to a genome-wide approach, where many or all common genetic variants are tested, with no (or fewer) prior assumptions [67].

In a typical Genome Wide Association Study (GWAS, e.g. [68]), experimental data is collected on the associations between several millions of SNPs and the disease under study. These associations are expressed as odds ratios (OR) calculated from SNP presence in patients relative to controls. The numbers of SNPs examined mean that large numbers of patient and control samples are needed to make the analysis useable and reliable. With even a few thousands of patients and controls, statistical probability thresholds must be in the order of 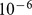 or less before significance can be established for an individual SNP. In addition, most studies do not make use of any previous knowledge that might have been published about particular genes and the disease.

Working with the WHO's cancer epidemiology lab in Lyon, France (IARC, <6>), we have developed a GWAS method that consistently ranks susceptibility SNPs significantly higher [69], [70]. This method – Adjusting Association Priors with Text (AdAPT) – searches research paper abstracts for prior knowledge on each SNP. This prior knowledge is in the form of counts of terms related to the disease under study, in papers that discuss genes in the same region as the SNP. For a GWAS of a particular disease, domain experts define a list of terms associated with the disease. For example, terms for anatomical sites and environmental factors associated with the disease may be selected. For each SNP, we find research papers related to genes in the same region as that SNP, and find the frequency of each term in those papers.

These lexical counts are combined with the experimental OR in a Bayesian model – Bayesian False Discovery Probability (BFDP [71]). For each SNP, the OR is used to calculate the posterior probability, and the lexical counts are used to calculate the prior probability. Experimental results for SNPs will be given an increased relevance where there is an increased frequency of search terms associated with the SNP. For example, we could analyse the results of a GWAS on lung cancer patients with AdAPT, using “smoking” as one of our search terms. Research papers that mention that a gene has been associated with the buzz experienced on smoking will be taken into account, when calculating the relevance of experimental results about SNPs in the region of this gene.

Such prior knowledge about genes is buried in the text of scientific papers, and so to make use of it in BFDP we use text mining to find those papers that discuss particular genes, diseases, anatomical loci, drugs and so on. Initial post-hoc experiments with historical data [72] demonstrated that the technique could have been used to find several SNPs associated with lung cancer. One SNP, for example, was ranked 124th using OR alone. With BFDP and text mining, it was ranked 10th and would have been considered highly relevant for further study. This gene, along with several others, is shown in Table 1, where it can also be seen that using the AdAPT method makes rankings much more robust to a reduction in the amount of data used. A similar effect was found when examining a gene involved in several mechanisms relevant to kidney cancer. Typically, the technique requires half the data used in a typical GWAS to achieve the same results (which implies a possible cost saving of 50% on wet lab work).

**Table 1:** Comparison of P-Value and BFDP ranking.

SNP ID	Locus	Proportion of data samples	P-value		BFDP	
			Rank	Power	Rank	Power
rs1051730	15q25.1	100%	2	-	2	-
		75%	10	80%	8	81%
		50%	959	17%	793	18%
rs2736100	5p15.33	100%	77	-	8	-
		75%	2359	4%	222	31%
		50%	17989	3%	1350	16%
rs3117582	6p22.33	100%	124	-	10	-
		75%	2717	6%	184	35%
		50%	20033	3%	1038	13%
rs401681	5p15.33	100%	74	-	6	-
		75%	2775	8%	249	32%
		50%	25446	2%	1866	10%
rs4324798	6p22.1	100%	4	-	4	-
		75%	844	25%	545	28%
		50%	7495	3%	6178	3%
rs8034191	15q25.1	100%	1	-	1	-
		75%	4	87%	3	89%
		50%	502	24%	435	28%

By adding prior knowledge using the AdAPT method, genes robustly implicated in lung cancer are shown to rank more highly than based on p-value alone. This means that they could have been flagged for further investigation sooner, had the method been used.

More recently, we have applied the technique to new data. A gene involved in the regulation of alcohol metabolism was poorly ranked for head and neck cancer using OR alone, but highly ranked when BFDP and text mining were used. Based on this re-ranking, the gene was studied further and has now been shown to have an association with head and neck cancer [70].

The AdAPT method was motivated by the fact that a large proportion of highly ranked, yet statistically insignificant, SNPs in GWAS studies reside near potential candidate genes. GATE was used to provide a framework in which different methods of mining the literature could be experimented with, from simple surface processing of text, to matching text against ontologies and terminologies such as those found in UMLS using MetaMap [28]. Search terms were indexed in GATE Mímir, which will enable future experiments combining prior knowledge in both text and in structured knowledge such as ontologies.

A public demonstration service of the text analysis system is available online, see <7>.

### Clinical Records Mining for Evidence-Based Medicine

SLaM, the South London and Maudsley Hospital, covers a population of 1.1 million across a large area of South London. Their mental health unit has 35,000 patients, whose treatment records are stored in an Electronic Health Record (EHR) system containing some 175,000 records. The EHR system supports 5,000 active users.

SLaM is host to the UK National Institute of Health Research Biomedical Research Center (BRC) for Mental Health. The BRC have built the largest mental health case register in Europe, using data extracted from the SLaM EHR. This case register is known as CRIS, Case Register Interactive Search system [73]. Data in CRIS is de-identified and indexed for search via a web interface and standard database query languages. Access to CRIS is restricted by an institutional policy framework.

The BRC performs a central research function for policy making (at both regional and national level) and medical audit (informing evidence-based and translational medicine). Typical research questions tackled by BRC epidemiologists might include:

- Is there a test for those with Alzheimer's disease that can show if drugs would be the best treatment?
- Do some drugs for schizophrenia affect physical health, e.g. diabetes?
- Do people's home living arrangements affect how long they spend as inpatients, receiving care in hospital wards?

BRC researchers use a variety of data sets and tools in their work, often linking and merging different data, and employing a wide variety of statistical analyses. CRIS is only one tool in this process, but a very useful tool in that it provides an unrivalled data set at the level of the individual patient and health care episode. CRIS contains much structured data from the EPR. In many cases, however, useful information is present only in the free text fields of CRIS, which contain a mixture of correspondence from SLAM clinicians to primary care physicians, and short notes made during clinical work. ‘Clinicians, and mental health clinicians in particular, are in love with free text’, notes Matthew Broadbent, BRC CRIS manager (during ‘GATE for Life Sciences: extracting information from electronic health records’, a talk at the GATE training course of May 17th 2011). CRIS contains some *11,000,000 free text field instances* in its records. Even though computer literacy is increasing amongst clinicians (partly as younger practitioners move upwards through the system), still it seems likely that this ‘love affair with free text will be almost impossible to break’, at least in the medium term. Medics often cite lack of time during clinical practice as a reason that large quantities of data that is highly significant for clinical practice is not present in the structured record at all. Additionally, the free text portion of the record contains letters to primary care physicians, and so has a legal status in the UK that is not afforded to the structured record. Examples of the value of the free text record over the structured record at SLaM include:

- smoking status is only ever recorded in the free text fields;
- some diagnoses are only present in the free text, e.g. 800 cases of Alzheimers were identified from a set of 4900 records, where the diagnosis was not recorded in the structured data;
- for a widely used score of cognitive ability (MMSE – see below), a query to the structured field returned 5700 hits; adding a keyword search over the free text fields returned an additional 48,750 hits.

Clearly, if the free text is ignored, researchers will miss a large portion of the data. Starting in 2010 the BRC began a programme of work with GATE to extract data from their free text records. The BRC uses GATE to create extraction pipelines for a variety of textual entities and events. The set of entities and events extracted are not fixed. They are shifting and evolving, as new research questions emerge, and as the possibilities of information extraction are explored by researchers. Specific pipelines are developed in response to the needs of individual research projects, although many find re-use in other projects. GATE is therefore seen as an additional research tool, rather than as a black box application that extracts a limited set of entities. The BRC sees GATE as an information extraction capability rather than as a single application: they use the GATE process as described above to develop each new application, making use of manual annotation facilities to create evaluation corpora, and GATE's quality control tools to measure progress. Each pipeline is developed through up to 6 iterations of definition, prototyping, and accuracy measurement. Applications in use include ones to extract patient smoking status, diagnosis, social care, level of education, and medications.

We describe one such application here, the extraction of Mini Mental State Examination (MMSE) results. MMSE is a test of cognitive ability, scored out of 30, and frequently used in cases such as memory loss or dementia. There are many occurences of MMSE reported in the CRIS free text data, for example ‘MMSE done on Monday, score 24/30’. The extraction task was to find MMSE assessments described in the text, together with their scores and dates. Complications in the extraction of this data include:

- date normalisation relative to proximate dates in the free text, or as a last resort the document instance date (e.g. what date does ‘Monday’ refer to in the above example?)
- conjunctions, negations, coordinations etc. (e.g. ‘patient X scored Y/30 in November then Z/30 in December’)

During development of the MMSE application, BRC decided to favour precision over recall for this task. The output of MMSE extraction is used to create MMSE time series from the multiple documents held for each individual patient, and they calculate that missing some occurences of MMSEs within these series does not negatively impact the research conclusions that they are drawing from the analyses, whereas false positives would be more problematic.

MMSE extraction task guidelines were written by clinical domain experts, and refined iteratively while using them for manual annotation of MMSE in example texts. The MMSE application was developed over four iterations. At the end of each iteration, the application was run over unseen evaluation texts. The annotations in these texts were then corrected by domain experts, and standard information extraction evaluation metrics used. Precision was used to give the proportion of the annotations created by the system that are correct, compared to the human sources. Recall was used to give the proportion of the human annotations that the system had found. (See e.g. [3] for a fuller explanation of these evaluation measures.) The corrected annotations were then made available, as development data for creation of the next iteration of the application. After four iterations, 224 documents containing 270 MMSE events had been used. Evaluation against the final set of unseen evaluation texts gave a precision of 0.89, and a recall of 0.94 in correspondence texts, and a precision of 0.85 and a recall of 0.85 in short note texts. The final application was also evaluated against a set of 1456 manually extracted MMSE events from 6236 documents. This evaluation gave a precision of 0.83 for the MMSE score, and 0.79 for the MMSE date. In the case of MMSE, and of GATE applications for the extraction of other events in CRIS text, it has been possible to attain an accuracy that is sufficient to support drawing conclusions for policy and audit purposes.

When the MMSE application was run over the full CRIS data set, a post-processing step was added that makes a number of heuristic sanity checks (using domain rules) against the structured data and filters out problematic results from the extraction engine. For example, MMSEs are always scored out of 30 – so a numerator of more than 30 or a denominator that isn't 30 indicates an error (either in the notes themselves or in the extraction components). Similarly, a date given for an examination that is in the future relative to the parent record date must be incorrect. Deduplication may also be performed.

Results from running the MMSE application over the full CRIS data set illustrated a further point. The MMSE extraction system found 58,000 MMSE scores out of 48,000 relevant free text documents. After post-processing, 35,000 instances remained. Following further data checking (including comparison between the structured records and the free text extraction data), and contrary to initial expectations, samples of data suggested that the MMSE data being extracted from the free text was *more* accurate than the structured data (i.e. the coding quality of the database MMSE data can be low). It appears that in this case, the structured record may be less accurate than that recorded in the free text, and where a high-precision extraction system can be built, even the text analysis results may be more accurate than the structured data.

For the MMSE extraction task, computational resources deployed were on the order of 40 processor nodes for 24 hours (running as a batch process of 11,000,000 XML files dumped from the database). The individual jobs are run using GATE Cloud Paralleliser, the server-level infrastructure from GATE Cloud as described above.

### Drug-Related Search in Patent Data

Our third (and last) example is motivated by three interlocking concerns. First, patents are currently a relatively opaque and under-exploited resource for scientific exploitation. On the one hand a globally significant amount of research work is encapsulated in patent documents (and in many cases these documents are the only source of publication, due to commercial confidentialty constraints [74]), on the other hand access to and analysis of the patent record is typically problematic and partial [75]. The exposure of quantitative biomedical data derived from patents of the type discussed below is one way to ameliorate this concern.

Second, search of high-value content is moving beyond ‘bag of words’ methods and towards semantic and conceptual query and navigation methods [76]. Patents are a valuable resource in many contexts, not least the pharmaceutical, and the provision of additional search modalities using biomedical taxonomic structures over patent data is in demand in a variety of life science contexts.

Thirdly, The text mining part of this picture is also likely to be applicable to a wide range of experiments where abstraction over the published research record can be used to adjust probabilistic models – such as the cancer epidemiology work reported above.

As part of a research programme on new methods for searching patent data [47], [77] we developed (in conjunction with partners) a semantic search capability that combines the Federal Drug Administration's (FDA) Orange Book <9> with UMLS (the Unified Medical Language System, <10>) terms. This section describes the data integration approach used and the search application constructed. This use case is representative of applications of text mining in life sciences where the objective is to support additional search modalities (for example, facetted or conceptual queries).

Advances in molecular biology and genetics are now commonly based on petabytes of raw genome and protein sequence data. In organising and interpreting these raw data there has been a parallel growth in life sciences literature, and in databases, taxonomies, ontologies, knowledge bases, and other types of knowledge source. With respect to *literature*, consider that MEDLINE, the primary life sciences abstract database, currently stands at 21 million abstracts, and is growing at the rate of 600,000 abstracts a year. With respect to *knowledge sources*, consider that there are currently over 1000 ontologies, data- and knowledge-bases in the life sciences, and that typical gene databases contain over 400 million triples when in RDF form (making them some of the largest single semantic data repositories available) [54].

A number of problems arise which a combination of data integration, information extraction and text mining can sometimes help solve. As it is now impossible to read all relevant literature in a sub-area, and difficult to search using traditional IR techniques, there is increasing demand for IR methods that integrate the various knowledge sources and literature, allowing novel experimental setups such as that described above. Several projects exist which attempt to provide ‘mashups’ of knowledge sources, and which link knowledge sources to semantic annotation of the life sciences literature. Some of these attempts have also led to proposals for standard approaches to the use of RDF in the life sciences.

GATE includes support for exploiting this type of structured data in several ways, including:

- GATE Embedded includes a simple API for accessing ontological data (represented in RDF or OWL). This API is very basic – it doesn't replace other more comprehensive efforts, but it does provide a level of functionality appropriate for text processing applications without the complexity that arises from comprehensive support of the relevant standards. See Chapter 14 of the GATE User Guide <11> [5] for more details.
- Similarly GATE Developer has simple visualisation and editing tools for working with ontologies.
- CREOLE (the GATE plugin set) includes a *Large Knowledge Base Gazetteer* for direct annotation of concept lexicalisations from the OWL store in text. See the GATE User Guide <12> for more information.

When working with life sciences data, we often use the Linked Life Data OWL repository (LLD, <13> from Ontotext <14> – see above), an RDF data repository that integrates around four billion statements from existing databases, taxonomies and ontologies. Using GATE's ontology tools and the LLD knowledge base we developed a rich search application for drug search over patent data, to which we now turn.

The core of the approach is to semantically annotate patents with references to drugs, their ingredients, the organisations that have developed them, their typical dosages and routes of administration, and so on. On top of the resultant semantic index (in GATE Mímir) we then expose structured and co-occurrence based visual retrieval interfaces.

An ontology was first created capturing the classes and relationships evident from the structure of the data about patented drugs present in the FDA's Orange Book. This ontology was then aligned with a basic upper level ontology to reuse named entity classes and relationships, some of which are directly applicable to the domain (organisation, person, document, patent, location, and their corresponding relationships). The two ontologies, thus aligned, served as the conceptual schema for transformation of the drug descriptions from their Orange Book form into instances in the semantic database. In this way, the semantic repository was populated with:

- drug instances with their corresponding names;
- active ingredients or chemical compounds;
- the different dosage forms and strengths of the ingredients;
- routes of administration;
- target;
- the patent applicant;
- approval and expiration dates.

A simple semantic annotation pipeline was developed recognising mentions of known drugs, ingredients, dosage forms, and others, in the patent documents. The annotations link these mentions to instances modeled in the semantic database. Additionally, bibliographic metadata was transformed into document level metadata and passed for indexing along with the textual content and the semantic annotations.

The retrieval and navigation capabilities were based on a unified semantic index back-end based on Mímir. The retrieval capabilities exposed through the visual interfaces include:

- Predefined structured queries: looking for drugs with a particular route of administration, or the drugs by a specific applicant, etc. These are expressed as path pattern restrictions on the graph of the semantic index and the results are either entities or documents referring to these entities.
- The class taxonomy of the FDA ontology can be browsed and examined.
- Co-occurrence based navigation and retrieval interfaces. The co-occurrence of entities in the same context is the key navigation and retrieval restriction paradigm in this case, exposed through facets listing entities from a particular class. The system can be tuned to show the entities in lexicographic order, or order based on their frequency in the current selection of patents.
- Traditional full text boolean search is also available, enriched with restrictions over metadata fields and document structure.
- Trend analysis is available based on analyzing how frequency of entity mentions changes through the different points in time associated with the documents. This results in interactive timelines of entity popularity on a previously selected set of patents, time interval and display granularity. Thus, one can examine how, for example, the frequency in patent applications of references to ibuprofen and aspirin change through time. The different points forming the trend graphic lead directly to temporally restricted document sets forming the corresponding frequency of reference.

**Figure 7:**
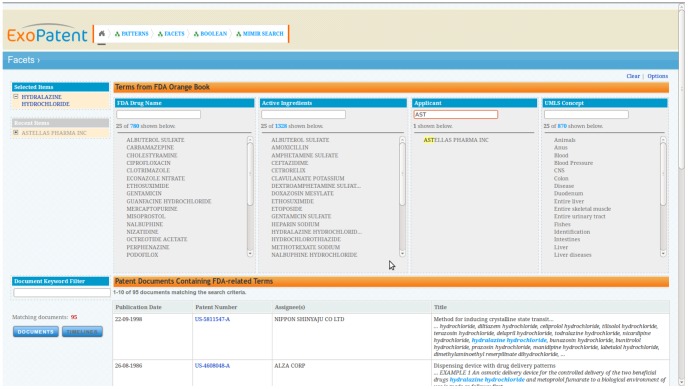
Co-occurence search. Faceted search allows users to apply multiple filters – here we have selected Hydralazine Hydrochloride as an Active Ingredient and started typing ‘AST’ in the Applicant column.

For example, the co-occurence faceted search interface (where each column represents a different semantic type extracted from the documents) is shown in Figure 7.

A public example service is available online at <16>.

## Availability and Future Directions

Dataset S1 bundled with this paper contains a distribution of GATE; dataset S2 contains the GWAS system described above.

GATE Developer and GATE Embedded are available under the Lesser GNU licence (LGPL, <17>). To download GATE Developer/Embedded, see <18>. The software will run anywhere that supports Java 6 or later, including Linux, Mac OS X and Windows platforms. We don't run tests on other platforms, but have had reports of successful installs elsewhere. Documentation includes a 650 page User Guide <19> [5] and thousands of pages of API and other documentation <20>.

GATE Teamware is available under the Afero GNU licence (AGPL, <21>) and on Amazon's server farms via GATE Cloud.

GATE Cloud is available online, see <3>. It is a simple matter to access the software as a service, and set up a project using the ready-made analysis services or run your own custom analysis pipelines.

GATE Mímir is available under the Afero GNU licence (AGPL, <21>). It is downloadable from SourceForge <22> and available as a service on GATE Cloud. Documentation is available in the form of a Users' and Implementors' Guide <23>.

Future development of GATE is driven by its user and developer community. New members can join this community via the mailing list, Facebook or LinkedIn groups. The software is hosted on SourceForge <24> where users may report bugs, request features and contribute patches. For those with a track record of contributing good code to the project, committer privileges are granted, allowing direct contribution to the codebase.

An easy way to add new functionality to the project and share it with other users is to make a plugin. GATE Developer/Embedded provides a flexible structure where new resources can be plugged in very easily. Full details of how to go about making and contributing a plugin can be found in Chapter 12 of the User Guide <25>.

## Links

<1> http://gate.ac.uk/

<2> http://gate.ac.uk/gate/doc/plugins.html

<3> http://gatecloud.net/

<4> http://ghr.nlm.nih.gov/

<5> http://mg4j.dsi.unimi.it/

<6> http://www.iarc.fr/

<7> http://services.gate.ac.uk/lld/gwas/service/

<8> http://www.slam.nhs.uk/research/biomedical-research-centre/brc-home

<9> http://www.accessdata.fda.gov/scripts/cder/ob/default.cfm

<10> http://www.nlm.nih.gov/research/umls/

<11> http://gate.ac.uk/userguide/chap:ontologies

<12> http://gate.ac.uk/userguide/sec:gazetteers:lkb-gazetteer

<13> http://linkedlifedata.com/

<14> http://www.ontotext.com/

<15> http://linkedlifedata.com/sources/

<16> http://exopatent.ontotext.com/

<17> http://www.gnu.org/licenses/lgpl.html

<18> http://gate.ac.uk/download/

<19> http://gate.ac.uk/userguide/

<20> http://gate.ac.uk/documentation.html

<21> http://www.gnu.org/licenses/agpl.html

<22> http://gate.ac.uk/family/mimir.html

<23> http://gate.svn.sourceforge.net/svnroot/gate/mimir/trunk/doc/mimir-guide.pdf

<24> http://sourceforge.net/projects/gate/support

<25> http://gate.ac.uk/userguide/chap:development

<26> http://pages.cs.wisc.edu/bsettles/abner/

<27> http://lexsrv3.nlm.nih.gov/LexSysGroup/Projects/gSpell/current/GSpell.html

<28> http://vega.soi.city.ac.uk/~abdy181/software/

<29> http://www.nactem.ac.uk/genia/

<30> http://mutationfinder.sourceforge.net/

<31> http://pingu.unige.ch:8080/NormaGene/

<32> http://eagl.unige.ch/BiTeM/

<33> http://metamap.nlm.nih.gov/

<34> ftp://ftp.ncbi.nlm.nih.gov/pub/tanabe/AbGene/

<35> http://www.seas.upenn.edu/~strctlrn/BioTagger/BioTagger.html

<36> http://alias-i.com/lingpipe/

<37> http://opennlp.apache.org/

<38> http://uima.apache.org/

<39> http://gate.ac.uk/family/process.html

<40> http://gatewiki.sf.net/

<41> http://www.semanticsoftware.info/organism-tagger

<42> http://gate.ac.uk/teamware/

<43> http://www.ir-facility.org/

## Supporting Information

Dataset S1 **GATE software.** Dataset S1 bundled with this paper contains a distribution of GATE (or see http://gate.ac.uk/download/).
(TGZ)

Dataset S2 **GWAS AdAPT software.** Dataset S2 contains the GWAS Adjusting Association Priors with Text (AdAPT) software.
(TGZ)
