# Supplementary material for: Getting More Out of Biomedical Documents with GATE's Full Lifecycle Open Source Text Analytics
Source: PLoS Comput Biol. 2013 Feb 7;9(2):e1002854. doi: 10.1371/journal.pcbi.1002854 (PMC3567135; doi:10.1371/journal.pcbi.1002854)
Supplement: Dataset S1 — GATE software. Dataset S1 bundled with this paper contains a distribution of GATE (or see http://gate.ac.uk/download/). (TGZ) [file pcbi.1002854.s001.tgz › plos-gate/src/gate/resources/splash.html]

**Hamish Cunningham,
Valentin Tablan,
Kalina Bontcheva,
Diana Maynard,
  
Niraj Aswani,
Ian Roberts,
Thomas Heitz,
Angus Roberts,**
  
Adam Funk,
Yaoyong Li,
Wim Peters,
Horacio Saggion,
  
Genevieve Gorrell,
Suman Aswani,
Danica Damljanovic,
Milan Agatonovic,
  
Sandra Szasz,
Marin Dimitrov,
Mike Dowman,
Andrew Golightly,
  
Mark Greenwood,
Eric Sword,
Oana Hamza,
Mark Hepple,
Fang Huang,
  
Kevin Humphreys,
Angel Kirilov,
Atanas Kiryakov,
Lucy Moffatt,
  
Julien Nioche,
Mark Leisher,
Dimitar Manov,
Damyan Ognyanoff,
  
Tamara Polajnar,
Borislav Popov,
Andrey Shafirin,
James Sun,
  
Cristian Ursu,
Milena Yankova,
Andrew Borthwick,
René Witte,
  
Johann Petrak,
Georgi Georgiev,
Marin Nozchev,
Ekaterina Stambolieva,
  
Ekaterina Mihajlova,
Philip Alexiev,
Iavor Jelev,
Brian Davis,
  
Erik Graf,
Sebastiano Vigna.
