# Supplementary material for: Getting More Out of Biomedical Documents with GATE's Full Lifecycle Open Source Text Analytics
Source: PLoS Comput Biol. 2013 Feb 7;9(2):e1002854. doi: 10.1371/journal.pcbi.1002854 (PMC3567135; doi:10.1371/journal.pcbi.1002854)
Supplement: Dataset S2 — GWAS AdAPT software. Dataset S2 contains the GWAS Adjusting Association Priors with Text (AdAPT) software. (TGZ) [file pcbi.1002854.s002.tgz › plos-gate-gwas/docs/notes.html]

GWAS Notes


# GWAS Notes

- The old version reports different p-values because it was getting the wrong value from the binding set
- The default LLD location is http://gate.ac.uk/lld/openrdf-sesame/ if you need to change this then set
  the bk.endpoint system property to point to the correct URL
