# Supplementary material for: Getting More Out of Biomedical Documents with GATE's Full Lifecycle Open Source Text Analytics
Source: PLoS Comput Biol. 2013 Feb 7;9(2):e1002854. doi: 10.1371/journal.pcbi.1002854 (PMC3567135; doi:10.1371/journal.pcbi.1002854)
Supplement: Dataset S2 — GWAS AdAPT software. Dataset S2 contains the GWAS Adjusting Association Priors with Text (AdAPT) software. (TGZ) [file pcbi.1002854.s002.tgz › plos-gate-gwas/docs/todo.html]

GWAS To Do List


# GWAS To Do List

- Change domain objects to support keyword groups **[DONE]**
- Update views to allow display/editing of keyword groups **[DONE]**
- Update Java code to support keyword grouping **[DONE]**
- remove the idea of BFDP from the service as we may want to support multiple metrics or just the prior search
- check if grouping form items are always correctly ordered
