# Supplementary material for: Getting More Out of Biomedical Documents with GATE's Full Lifecycle Open Source Text Analytics
Source: PLoS Comput Biol. 2013 Feb 7;9(2):e1002854. doi: 10.1371/journal.pcbi.1002854 (PMC3567135; doi:10.1371/journal.pcbi.1002854)
Supplement: Dataset S2 — GWAS AdAPT software. Dataset S2 contains the GWAS Adjusting Association Priors with Text (AdAPT) software. (TGZ) [file pcbi.1002854.s002.tgz › plos-gate-gwas/docs/ideas.html]

GWAS Ideas


# GWAS Ideas

- Can we use the SNP db in NCBI Entrez which will give us links to pubmed etc free!
- Do we just want to limit to human or any organisim for which there is data?
- We need to have a way of storring the service results as a stream so it can be stop/started
